# Effect of nitrogen starvation on desiccation tolerance of Arctic *Microcoleus* strains (cyanobacteria)

**DOI:** 10.3389/fmicb.2015.00278

**Published:** 2015-04-08

**Authors:** Daria Tashyreva, Josef Elster

**Affiliations:** ^1^Centre for Polar Ecology, Faculty of Science, University of South BohemiaČeské Budějovice, Czech Republic; ^2^Department of Botany, Faculty of Science, University of South BohemiaČeské Budějovice, Czech Republic; ^3^Institute of Botany, Academy of Sciences of the Czech RepublicTřeboň , Czech Republic

**Keywords:** cyanobacteria, desiccation tolerance, viability, nitrogen starvation, fluorescence staining, SYTOX Green, CTC dye

## Abstract

Although desiccation tolerance of *Microcoleus* species is a well-known phenomenon, there is very little information about their limits of desiccation tolerance in terms of cellular water content, the survival rate of their cells, and the environmental factors inducing their resistance to drying. We have discovered that three *Microcoleus* strains, isolated from terrestrial habitats of the High Arctic, survived extensive dehydration (to 0.23 g water g^-1^ dry mass), but did not tolerate complete desiccation (to 0.03 g water g^-1^ dry mass) regardless of pre-desiccation treatments. However, these treatments were critical for the survival of incomplete desiccation: cultures grown under optimal conditions failed to survive even incomplete desiccation; a low temperature enabled only 0–15% of cells to survive, while 39.8–65.9% of cells remained alive and intact after nitrogen starvation. Unlike *Nostoc*, which co-exists with *Microcoleus* in Arctic terrestrial habitats, *Microcoleus* strains are not truly anhydrobiotic and do not possess constitutive desiccation tolerance. Instead, it seems that the survival strategy of *Microcoleus* in periodically dry habitats involves avoidance of complete desiccation, but tolerance to milder desiccation stress, which is induced by suboptimal conditions (e.g., nitrogen starvation).

## Introduction

Terrestrial cyanobacteria are often considered to be desiccation tolerant organisms. Some taxa of cyanobacteria have evolved a remarkable ability to resist desiccation stress (Caiola et al., 1996; Potts, 1999). This ability has allowed them to colonize the most hostile places on Earth.

Numerous studies have uncovered that cyanobacteria cope with desiccation stress through a complex of physiological, biochemical, structural, and morphological adaptations. Mechanisms contributing to this include: modifying the structure and composition of cell envelopes (Caiola et al., 1996), decreased respiration (Potts, 1994), down-regulation of photosynthesis (Harel et al., 2004), producing enzymes eliminating reactive oxygen species (Chen et al., 2012), accumulating sugars which stabilize the lipid membranes (Hershkovitz et al., 1991; Sakamoto et al., 2009; Klähn and Hagemann, 2011), secreting extracellular polysaccharides that serve as a physical barrier during desiccation that absorb and retain moisture (Hill et al., 1997; Tamaru and Takani, 2005), synthesizing UV-absorbing and sun-screening pigments (Roos and Vincent, 1998; Potts, 1999; Gao and Ye, 2007), and the presence of multiple copies of the genome together with an efficient DNA reparation system (Ehling-Schulz and Scherer, 1999; Potts, 1999).

The closely related genera *Phormidium* and *Microcoleus* (Oscillatoriales) are among the most frequently recorded cyanobacterial genera in hot and cold deserts worldwide (Vincent, 2000; Wynn-Williams, 2000), and have often been mentioned as desiccation tolerant organisms (Davey, 1989; Hershkovitz et al., 1991; Hawes et al., 1992; Harel et al., 2004; Šabacká and Elster, 2006; Chen et al., 2012; Olsson-Francis et al., 2013). The taxonomy of Oscillatoriales has been recently revised to include some *Phormidium* species within the *Microcoleus* genus (Strunecký et al., 2013). Therefore, in this manuscript we consider *Phormidium* and *Microcoleus* as synonyms, and refer to their original names used in publications.

Some studies have suggested that *Phormidium* might respond to drying differently than the extremely desiccation tolerant and well-studied *Nostoc* and *Chroococcidiopsis*. These latter two genera were found to withstand regular drying-rewetting cycles, tolerate rapid water loss to nearly zero water content (Caiola et al., 1996; Tamaru and Takani, 2005), preserve the structural integrity of their cell structures after many years of storage in a dry state (Potts, 1996; Billi, 2008), and resume respiration and photosynthesis within minutes after rewetting (Davey, 1989; Wynn-Williams, 2000). In contrast to *Nostoc* colonies, *Phormidium*-dominated mats from the Antarctic showed a very slow recovery from extreme desiccation; the population survived due to migration and the multiplication of a few surviving middle-layer trichomes (apparently partially hydrated), rather than recovering the bulk biomass (Hawes et al., 1992). Hot desert dwelling *Oscillatoria* and *Microcoleus* species also demonstrated desiccation avoidance behavior as they actively migrated to the soil crust surface when water became available and retreated to the subsurface under water limitation (Pringault and Garcia-Pichel, 2004; Rajeev et al., 2013).

The few mechanisms discovered of *Phormidium/Microcoleus* desiccation tolerance include accumulating trehalose (Hershkovitz et al., 1991; Chen et al., 2012), secreting exopolysaccharides (Chen et al., 2012), stabilizing the photosynthetic apparatus (Harel et al., 2004), and accumulating UV-protecting pigments (Quesada and Vincent, 1997). A recent study on the desert crust-forming cyanobacterium *Microcoleus* *vaginatus* reported the expression of genes involved in the oxidative and osmotic stress response, the desaturation of membrane lipids, and the production of EPS at the onset of desiccation. Rehydration activated the genes responsible for cell signaling and DNA repair followed by upregulation of anabolic pathways (Rajeev et al., 2013).

Taken together, it is likely that *Phormidium*/*Microcoleus* evolved a combined strategy for surviving dry periods including both avoidance and partial tolerance to desiccation, rather than the ability to tolerate complete desiccation. However, it is not known whether desiccation tolerance is their constitutive trait as in some groups of mosses (Oliver et al., 2005), or if it develops under particular conditions (e.g., suboptimal light and temperature, osmotic stress, or nutrient starvation), as in many species of yeasts and bacteria (Morgan et al., 2006). While some of the mechanisms have been described, there is very little information about their limits of desiccation tolerance in terms of water content in dry cells, the survival rate of cells, damage that cells sustain upon desiccation, and rehydration, and environmental factors inducing their resistance to drying.

In many previous studies that have addressed desiccation tolerance of *Phormidium*/*Microcoleus*, the conditions of desiccation, water content in dried material, and methods for quantification of viable cells were often not described. Most of the studies evaluated the survival and stress response of *Phormidium* and *Microcoleus* at the population level, e.g., ‘bulk’ measurement of respiration/photosynthesis measured by oxygen evolution/uptake, recovery of photosynthesis, or growth tests (Davey, 1989; Hawes et al., 1992; Chen et al., 2003; Harel et al., 2004; Šabacká and Elster, 2006; Rajeev et al., 2013). For instance, such an approach often overlooks the number of cells that survive and their physiological state upon rehydration. A decrease in respiration or/and photosynthesis intensity upon rehydration, for example, may be attributed to a reduction of those functions in every cell, complete inactivation of a subpopulation while the others remain fully active, or to the differential loss of these in a few subpopulations. The importance of studying microbial populations at the single-cell level has often been stressed in recent years (Davey and Winson, 2003; del Giorgio and Gasol, 2008; Lidstrom and Konopka, 2010; Tashyreva et al., 2013).

The investigation of desiccation tolerance of filamentous cyanobacteria is generally complicated by the structure of the populations they form: cultures form tight colonies during standard cultivation in a liquid medium (e.g., in Erlenmeyer flasks). The conditions across such a colony can be markedly different in terms of light spectrum and intensity, nutrient availability, and concentration of cell metabolites. In addition, cultivation on agar plates generates a water content gradient, under which the filaments on the top of a biofilm are directly exposed to air. This approach generates physiologically heterogeneous populations, and, in addition, cannot ensure uniform drying of such a colony/biofilm.

In order to resolve the above-mentioned methodological complications, we employed cultivating cyanobacteria in thin biofilms on glass slides immersed into dishes with a liquid medium. Such a cultivation method provides significantly more homogeneous conditions in comparison to traditional cultivation methods, making it possible to vary only one of the cultivation parameters by placing glass slides into different conditions with the other conditions remaining constant. Drying the thin biofilms helped to eliminate the development of desiccation tolerance directly induced by slow dehydration of a thick layer (Hershkovitz et al., 1991; Chen et al., 2012). Detecting desiccation survivors and investigating some of their cellular function was carried out by direct cell counts in combination with staining them with three fluorescent dyes to visualize the presence, location and shape of nucleoids, track membrane integrity, and detect respiration.

For our experiments, we selected three strains of *Microcoleus* inhabiting terrestrial habitats in the Arctic. The strains were isolated from ephemeral melt water streams and pools that often become completely dry and frozen in late summer.

In this study, we endeavored to determine: (1) whether desiccation tolerance is a constitutive property or if it is inducible by suboptimal conditions, i.e., low temperature and nitrogen depletion, (2) whether the strains are able to tolerate complete desiccation defined as water content below 0.1 g H_2_O g^-1^ dry biomass (Alpert, 2005) and/or incomplete (85% RH) desiccation regimes, and (3) which proportion of cells survives desiccation and what their physiological state upon rehydration is.

## Materials and Methods

### Cyanobacterial Strains

The experiments were conducted with strains *Microcoleus* sp. 816 CCALA (previously *Phormidium* cf.* autumnale*) isolated from a stream in the vicinity of a glacial moraine (Northern Sweden, Lapland, Abisko, 69∘21′N 18∘49′E); *Microcoleus vaginatus* 858 CCALA (previously *Phormidium* sp.) isolated from a small pool in a moraine (Svalbard archipelago, 77∘00′N 15∘20′E); and *Microcoleus* sp. 845 CCALA (previously *Phormidium* sp.) isolated from a stream with moss carpets (Svalbard archipelago, 77∘00′N 15∘20′E). All strains (isolated by Šnokhousová *et* Elster) are currently maintained in the Culture Collection of Autotrophic Organisms (CCALA), Institute of Botany, Academy of Sciences of the Czech Republic, with a taxonomical revision of these strains carried out by Strunecký et al. (2013). The strains were previously shown to be non-diazotrophic by the acetylene reduction method (unpublished data).

### Cultivation

Cyanobacterial cultures were pre-cultivated for 15 days in Erlenmeyer flasks in liquid BG-11 medium (Rippka et al., 1979) at +20∘C and a continuous photon flux density of 70 μmol m^-2^ s^-1^ (white light). The biomass was harvested and used as an inoculum for subsequent cultivation in biofilms.

A piece of the biomass was smeared over both sides of a glass microscope slide (76 mm × 26 mm); the filaments readily attached to the glass surface. Six glass slides were placed in an upright position in a rectangular glass dish (13 cm × 10 cm), and kept upright with a plastic holder. The dishes were filled with BG-11 medium so that it entirely covered the slides, and closed with a transparent lid, allowing gas exchange in a similar way as a Petri dish. The medium was continuously mixed with a magnetic stirrer at a low frequency (Topolino, IKA). The light source was located over the dishes, and light from the bottom was reflected with aluminum foil placed under the dishes. After 2 weeks of cultivation, half of the cultural medium was replaced with fresh BG-11 medium.

Previous studies have suggested that cyanobacteria are psychrotolerant but not psychrophilic (Tang et al., 1997). Although cyanobacteria in the Polar Regions are often subjected to high solar irradiances, it is known that low-light conditions are preferable for the growth of cyanobacteria (Sinetova et al., 2012; Jodłowska and Śliwińska, 2014). In our experiments (unpublished data), we found that two polar strains of *Microcoleus* yielded the highest biomass and chlorophyll a content at +20∘C and 70 μmol m^-2^ s^-1^ in full BG-11 medium. Therefore, we consider these light and temperature conditions to be optimal.

After 18–30 days of cultivation, biofilm samples from each of the six slides were collected for microscopic examination in order to study their morphology and viability. The biomass was considered suitable for subsequent experiments if the cells were of intense blue–green color, with well-pronounced thylakoids, uniform in morphology, having evidence of cell fission, lacked any visible cell inclusions, and containing only a small number of dead or decaying cells. The homogeneity of these cultures, in terms of cell viability and respiration activity, was tested with multicolor fluorescence staining (see below).

### Induction of Desiccation Resistance (Pre-desiccation Treatment)

Two of the glass slides from each of the dishes were transferred into a dish filled with nitrogen deficient BG-11_0_ medium (standard BG-11 medium lacking NaNO_3_), and incubated at +20∘C and continuous light of 70 μmol m^-2^ s^-1^ for 2–3 weeks. Another two slides were kept in the original dish, which was placed at +4∘C (70 μmol m^-2^ s^-1^ of light), and incubated for a week. The biomass from the remaining two slides had no pre-desiccation treatment and was directly used in desiccation experiments. Hereafter, these will be referred to as ‘control’ or ‘optimally grown’ biomass.

### Desiccation and Rehydration

Desiccation of the samples was carried out in two regimes: complete drying over silica gel and incomplete drying at 85% RH at 20∘C. For both tests, several patches of cyanobacterial biofilm (ca. 1 cm × 1 cm) sampled throughout both sides of the two slides were placed in a drop of culture medium inside three Petri dishes, and spread over the surface so that no folds were formed. Any excess liquid was removed with sterile filter papers. The temperature and humidity were measured with a digital thermo-hydrometer (KlimaGuard, TFA, Germany). The device was calibrated over P_2_O_5_ (0% RH) and saturated solutions of LiCl (11.3% RH) and KCl (85% RH).

Complete desiccation was achieved by placing dishes with biofilms in a stream of sterile air for 15 minutes and their subsequent storage in a closed chamber over silica gel for 20 days at low light (<10 μmol m^-2^ s^-1^). The RH over the silica gel fluctuated from 10 to 13%. Partial drying was carried out by placing the dishes for 20 days at low light (<10 μmol m^-2^ s^-1^) in a closed chamber over a saturated solution of KCl, which kept the relative air humidity at a constant 85%.

The films were rehydrated with a drop of sterile distilled water for 20 min while protected from light. The rehydrated biomass formed a thick suspension after being detached from the glass surface. Half of this suspension was then transferred into an Eppendorf tube with BG-11 medium for fluorescence staining, while the other half was used for a growth test.

### Viability Tests

Cell viability and physiological activity were evaluated with fluorescence staining. SYTOX Green dye (Life Technologies, USA) was used to track damage to the plasma membrane, 5-cyano-2,3-ditolyl tetrazolium chloride, or CTC (Sigma-Aldrich Co., USA) was used to assess respiration activity, and 4′,6-diamidino-2-phenylindole, or DAPI (Life Technologies, USA) was employed to observe the presence, shape, and location of nucleoids. The samples were treated according to the staining protocol that we previously described (Tashyreva et al., 2013) with 1 μM SYTOX Green for 30 min, 4 mM CTC solution for 30 min, and 5 μg ml^-1^ DAPI for 15 min. All the samples were observed with standard light microscopy prior to staining with fluorescent dyes. According to the staining results, the cells were grouped into three categories: (i) live and intact: CTC and DAPI-positive, SYTOX Green-negative; (ii) injured: CTC, DAPI, and SYTOX Green-positive; (iii) dead: CTC-negative, SYTOX Green, and DAPI-positive, or all negative. The viability test was done prior to desiccation and 20 min after rehydration.

A growth test was carried out with samples that underwent desiccation in order to confirm the staining results. The non-stained half of the biomass suspension was spread onto a BG-11 agar surface, and cultivated for 3–5 weeks under the same conditions as in pre-cultivation. In order to track any hidden growth, the dishes were periodically observed under a microscope under transmitted light (magnification 200×). The results of the growth test were expressed as having a presence or absence of growth.

### Fluorescence Microscopy

An aliquot of the stained sample was placed between a glass slide and a 24 mm × 24 mm cover slip; the edges were sealed with nail polish to prevent water evaporation. An Olympus BX53 microscope equipped with a 100 W ultrahigh-pressure mercury arc lamp (Olympus) was used with 400× magnification. The optical system for fluorescence observations included four UIS2 fluorescence mirror units (excitation filter/emission filter/dichromatic mirror): U-FBWA cube for SYTOX Green (460–495 nm/510–550 nm/505 nm), and combined mirror units for DAPI (360–370 nm/460–510 nm/420 nm), CTC-formazan (425–445 nm/570–625 nm/455 nm) and phycobiliprotein (565–585 nm/600IF/595 nm) fluorescence observation. A U-FUN filter cube (360–370 nm/420IF nm/410 nm) was additionally employed for observing the fluorescence of the DAPI-stained polyphosphate inclusions.

### Cell Counts

The biomass for fluorescent staining prior to desiccation was randomly sampled from both sides of every two slides in each treatment. After the staining procedure, the biomass was used to prepare several microscopy slides, where 2–5 fields of view were photographed from each of them, resulting in a total of 15–20 fields of view observed per sample.

A series of several dark-field images were acquired to record the fluorescence of each of the signals required and a bright-field image was taken for total cell counts. The images were captured with an Olympus DP72 microscope digital camera (Japan). Each image within a series was divided into 12 squares by applying a grid in GIMP v.2.8 program, where all cells within 1–3 squares were counted and distributed among three groups according to the staining results. For each of the experiments, 1100–1400 cells were counted in total.

### Statistical Analysis

The effects of the treatment, strain, sampling time (i.e., before and after desiccation), and their interactions on the proportion of dead cells were tested by repeated measures analysis of variance (ANOVA) using S-plus ver. 4.5 (Statistical Sciences, 1999). The repeated-measure factor (the qualitative independent variable) was the within-subjects factor, while the dependent quantitative variable on which each participant (one replicate of a strain) measured was the dependent variable (in proportion of dead cells). Tests of normality and equality of variances were performed and the data were found to be non-normal. Therefore, the dependent variable (proportion of dead cells) was arcsin transformed before using ANOVA.

### Estimation of Water Content

Ten pieces of the biofilms of strains 816 and 845 CCALA were placed on thin squares of aluminum foil and desiccated in the same way as in the experiments. The samples were weighted on analytical-grade scales before and after oven drying for 5 h at 102∘C. The water content was expressed per unit of dry mass.

## Results

**FIGURE 1:**
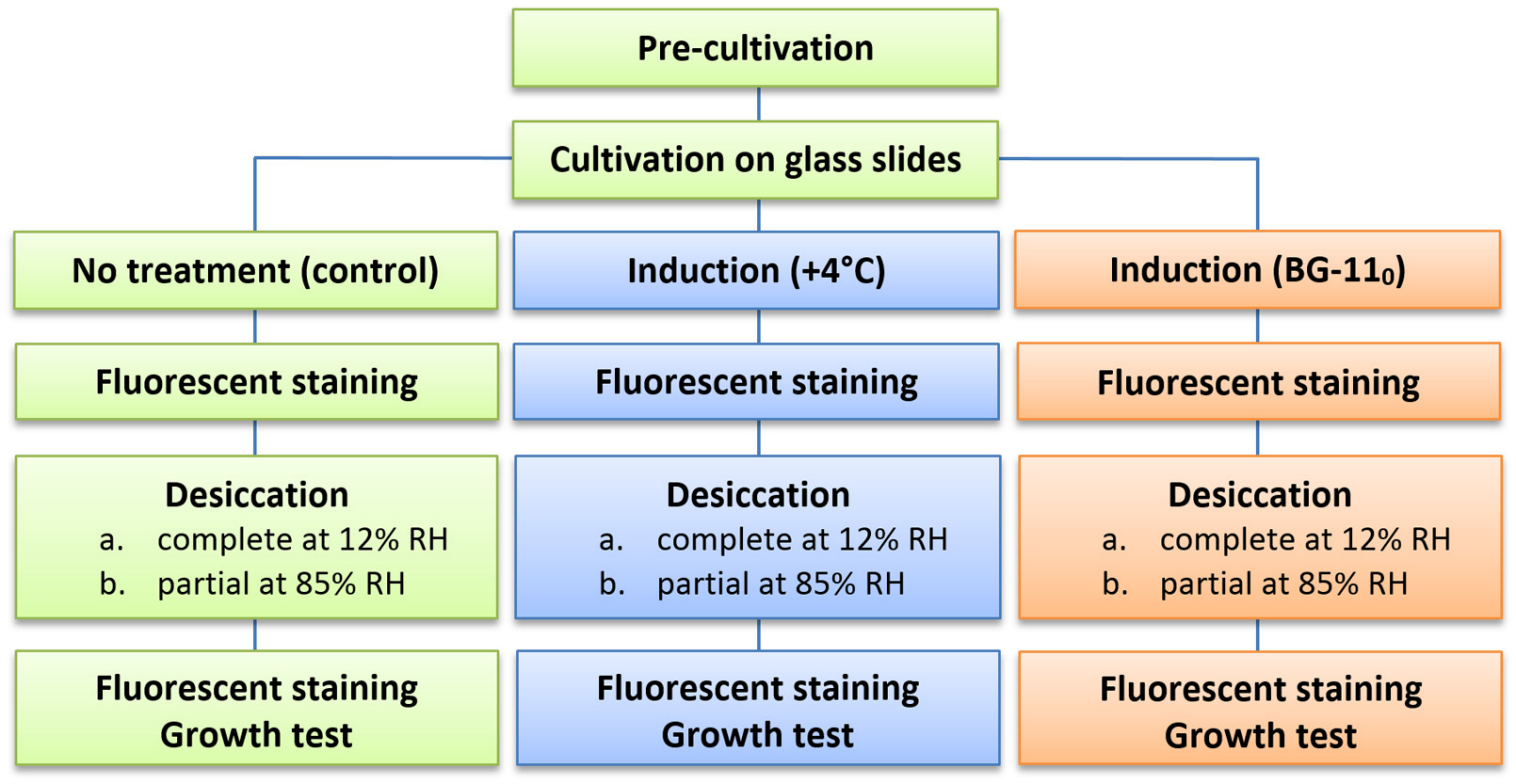
**Flow diagram of the experimental procedure for each of the strains**.

The general scheme of the experimental procedure is shown in **Figure 1**. All experiments were run in triplicate, i.e., pre-cultivation and cultivation in biofilms, subsequent pre-desiccation treatments, desiccation at both regimes, and viability tests were run three times separately for each of the strains.

.

**FIGURE 2:**
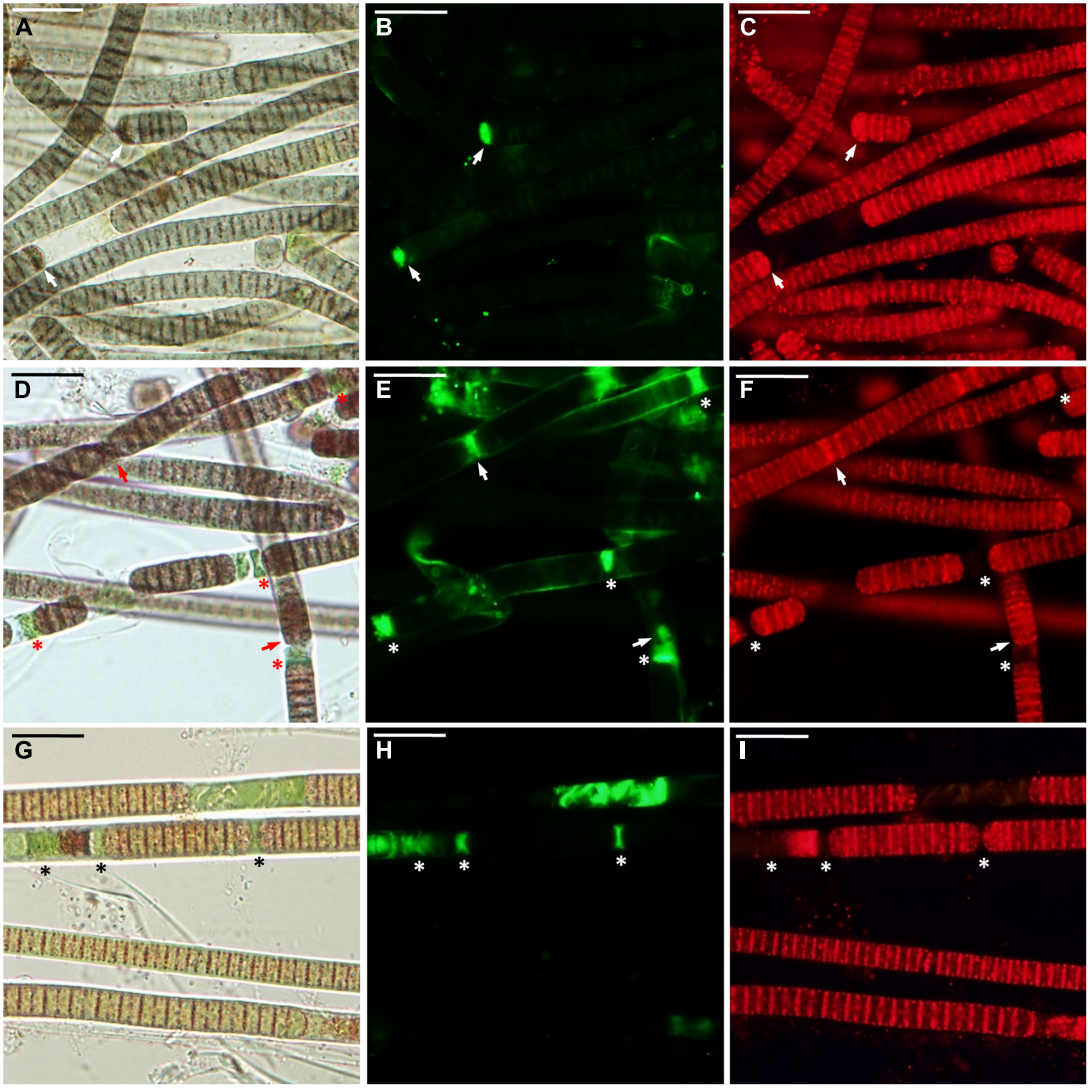
***Microcoleus vaginatus* 858 CCALA before desiccation. (A–C)** Culture grown under optimal conditions, viewed by light microscopy **(A)**, stained with SYTOX Green **(B)**, and CTC **(C)** fluorescent dyes; injured cells are marked with *arrows*. **(D–F)** Culture, kept at low temperature, viewed by light microscopy **(D)**, stained with SYTOX Green **(E)**, and CTC **(F)**; necridic (dead) cells are SYTOX Green-positive and CTC-negative (*asterisks*); the injured cells are both SYTOX Green and CTC-positive (*arrows*). **(G–I)** Nitrogen-starved culture viewed by light microscopy **(G)**, stained with SYTOX Green **(H)**, and CTC **(I)**; dead (in this case, decayed) cells are marked with *asterisks*. Scale bars are 20 μm.

### Morphology and Viability of Cells in Biofilms

Cyanobacterial cultures grown on glass slides formed thin biofilms, with loosely arranged filaments (Supplementary Figure S1). This cultivation regime provided consistent conditions for growth in terms of nutrient concentrations, light spectrum and intensity, and gas exchange.

**FIGURE 3:**
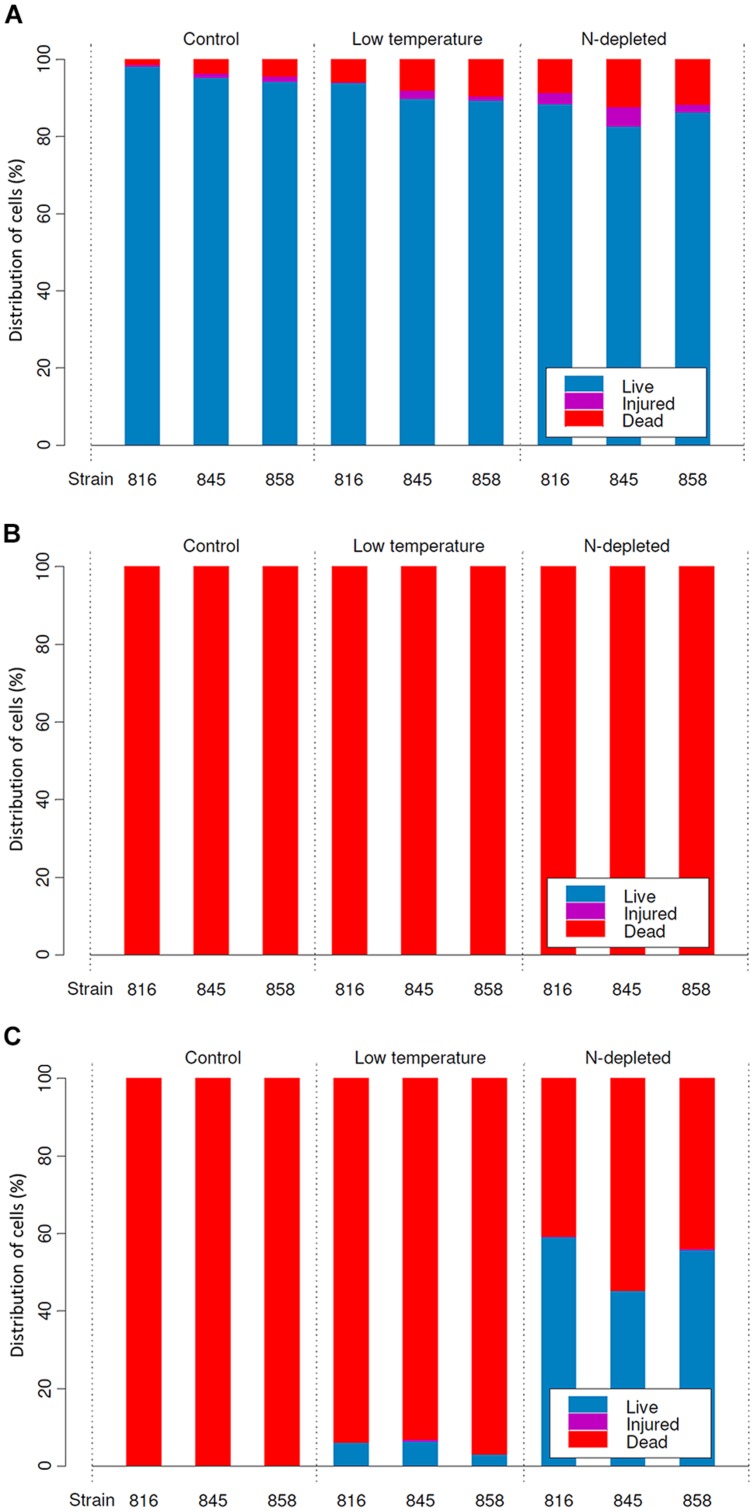
**Distribution of live, injured and dead cells in cultures (means) before desiccation (A), and after rehydration from complete (B), and incomplete (C) desiccation regimes**.

Under optimal conditions, the cells were uniform in size and morphology, had an intense blue–green color and fluorescence of phycobiliproteins, well-pronounced thylakoids, lacked cell inclusions, and were arranged in long filaments (**Figure 2A**). The viability of these cells was confirmed by the lack of SYTOX Green staining (**Figure 2B**) and the accumulation of numerous small CTC-formazan deposits within each cell (**Figure 2C**). The cultures had a low percentage of dead (0.9–4.6%) and injured cells (0–2%) in different replicates/strains (**Figure 3A**). Most of the injured and dead cells occurred at the polar ends of filaments, possibly because of mechanical disruption of filaments during the staining procedure (**Figure 2B**).

.

All the cultures kept for a week at +4∘C were well-pigmented, but variable to some extent in morphology and size. In most of the replicates, the cells had granulated cytoplasm apparently due to the accumulation of cyanophycin (irregularly shaped granules), and often formed necridic cells, resulting in the splitting of trichomes into short fragments (**Figure 2D**). There were higher numbers (**Figure 3A**) of injured (0–4.2%) and dead cells (2.4–13% in different replicates/strains). The dead cells mostly occurred singly or in rows within filaments and were often represented by necridic cells (**Figure 2E**). Injured cells, both SYTOX and CTC-positive, were often adjacent to necridic cells (**Figures 2E,F**).

Storing the cultures for 15–20 days in nitrogen-depleted medium led to a degradation of thylakoids, cell bleaching (**Figure 2G**), and a decomposition of phycobiliproteins, as was seen from the absence of their fluorescence in the red part of the spectrum. Despite this fact, the cultures maintained metabolic activity as was seen from the accumulation of CTC-formazan crystals in 87.4–93.5% of the cells (**Figures 2G,I**), of which 0.6–8.4% had permeabilized membranes (i.e., injured cells). Under nitrogen limitation, the quantity of dead cells (**Figure 3A**), including those with visibly deteriorated nucleoids, was the highest of all treatments (5.4–14.7%); they were scattered between filaments and across a sample without any obvious pattern (**Figures 2G–I**). Live, dead, and injured cells were morphologically similar, or, more often, dead cells appeared decayed (**Figures 2G–I**). Nucleoids were either unfolded or slightly condensed (data not shown). A proportion of cells accumulated polyphosphate deposits presumably, detected according to a shift in DAPI fluorescence from blue to yellow–green (data not shown, see Tashyreva et al., 2013). Distribution of live, injured, and dead cells were not significantly different among strains in each of the pre-desiccation treatments (see statistical comparisons at the end of results).

### Cell Viability after Complete Drying (12% RH)

**FIGURE 4:**
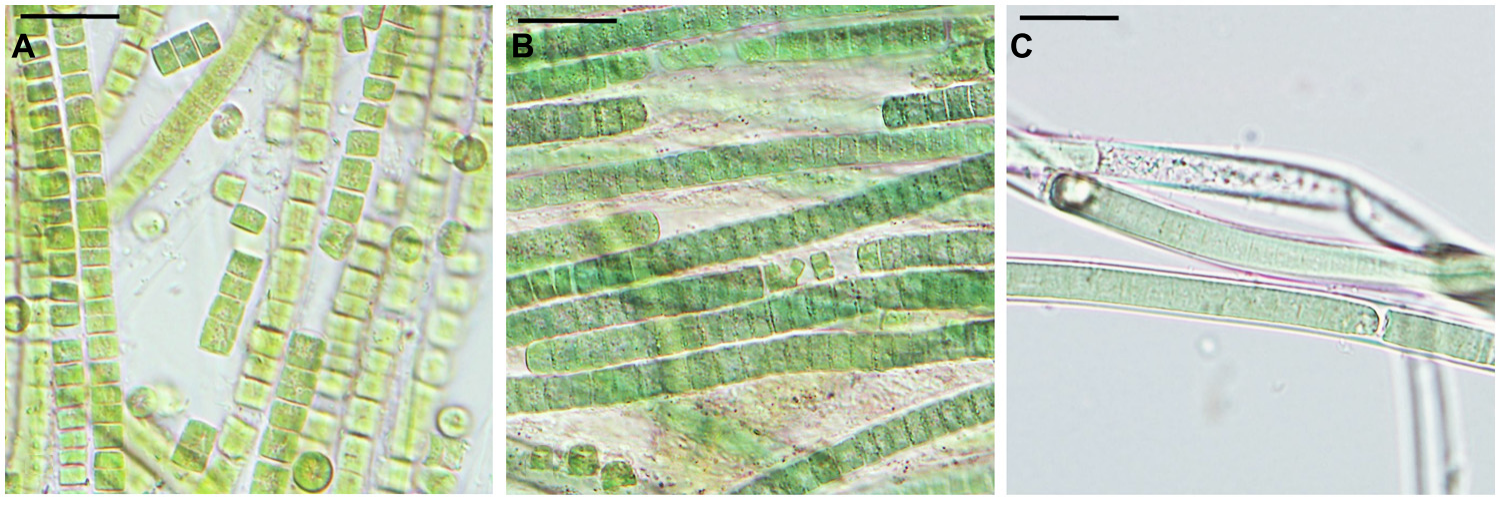
***Microcoleus vaginatus* 858 CCALA after rehydration from complete desiccation, viewed by light microscopy.** Cultures grown under optimal conditions **(A)**, and kept at low temperatures **(B)**, both containing filaments disintegrated into single cells; nitrogen-starved culture **(C)** with filaments enclosed in sheaths. Scale bars are 20 μm.

Samples that underwent a complete drying regime appeared dry after a few minutes under the stream of air. The samples contained 0.03 ± 0.001 g water g^-1^ dry mass (mean ± SD) after drying over silica gel for 2 weeks. No live or viable but injured cells were detected upon rehydration in any of the replicates grown under optimal (control) conditions (**Figure 3B**). In samples treated with low temperature and nitrogen depletion prior to drying, no viable cells were observed either (**Figure 3B**), despite the presence of sheaths in the nitrogen starved cultures (**Figure 4C**). In all treatments/replicates, the filaments started to disintegrate into single cells a short time after rehydration (usually within 1 h) followed by their quick decay (**Figures 4A,B**). Fluorescence staining (data not shown) revealed that all the cells were CTC-negative and SYTOX Green-positive, indicating the absence of respiration and damage to their plasma membranes. A small number of cells were both SYTOX Green and DAPI-negative, which indicated deterioration of intracellular components, including nucleoids. This staining pattern corresponded to the category of injured and inactive, or dead cells. The growth test showed consistent results – no growth was detected after 5 weeks of cultivation, and the biomass used as inoculum underwent lysis. No statistical analysis was applied to this group.

### Cell Viability after Incomplete Drying (85% RH)

**FIGURE 5:**
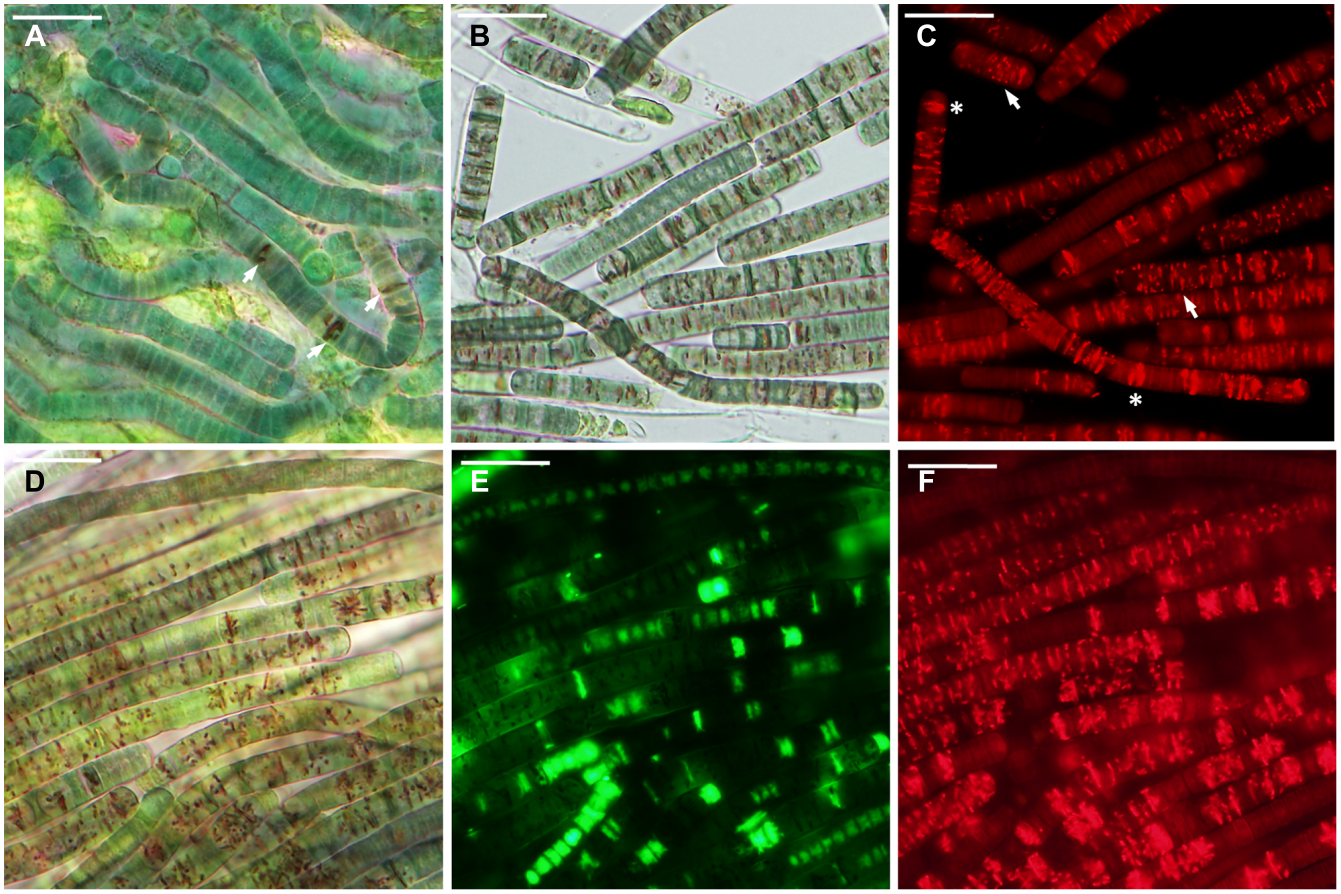
***Microcoleus vaginatus* 858 CCALA after rehydration from incomplete desiccation (85% RH). (A)** Culture grown under optimal conditions containing cells with a few large CTC-formazan crystals (*arrows*), which are visible under transmitted light as dark-red deposits. **(B,C)** Culture, kept at low temperature, viewed by light **(B)**, and fluorescence **(C)** microscopy; cells contain CTC-formazan crystals, which range from a few big ones (*asterisks*) to numerous small ones (*arrows*).** (D–F)** Nitrogen-starved culture, viewed by light microscopy **(D)**, and stained with SYTOX Green **(E)**, and CTC **(F)**; live cells are SYTOX Green-negative and CTC-positive; dead cells are SYTOX Green-positive and CTC-negative. Scale bars are 20 μm.

The samples contained 0.23 ± 0.01 g water g^-1^ dry mass (mean ± SD) after being stored over KCl solution for 2 weeks. In cultures grown under optimal conditions, only a few viable cells (5–20) per whole sample (i.e., millions of cells) were detected in some of the replicates, whereas others lacked any viable cells (**Figure 3C**). The absence of viable cells also proves that the drying treatment itself did not induce development of desiccation tolerance. Those solitary cells were scattered uniformly across the sample. They were SYTOX Green-negative and accumulated CTC-formazan deposits (data not shown). However, the deposits were only few and appeared much bigger in size (**Figure 5A**) compared to those in non-desiccated cells grown under optimal conditions (**Figures 2A,C**). A similar pattern of CTC-formazan deposition was observed in cells treated with sub-lethal concentrations of formaldehyde, possibly indicating cellular damage which cannot be tracked with SYTOX Green staining (Tashyreva et al., 2013). Apparently, such cells did not propagate because there was no evidence of growth, even after 5 weeks of cultivation.

Cultures that underwent low temperature treatment prior to desiccation showed complicated patterns of their desiccation response. No viable cells were detected upon rehydration in two of three replicates of strain 858 CCALA and in one of strains 845 and 816 CCALA. The biomass in the remaining replications contained 5–15% of viable cells clustered together (**Figure 3C**). The viable cells contained CTC-formazan crystals, which ranged from a few big ones to numerous small ones (**Figures 5B,C**), and were SYTOX Green-negative.

Nitrogen-depleted cultures showed the highest rate of desiccation survival. The proportion of viable cells was 39.8–51.3% for strain 845 CCALA, 41.2 to 65.9% for 858 CCALA, and 56.8 to 62.3% for strain 816 CCALA (**Figure 3C**). Fluorescence staining revealed that cells that survived desiccation resumed their metabolic activity (i.e., respiration) within minutes after rehydration; their CTC-formazan deposits ranged from a few big ones to numerous small crystals (**Figure 5F**). Those cells remained intact according to the absence of SYTOX Green staining (**Figure 5E**), contained unfolded nucleoids (stained with DAPI, data not shown), and were not notably morphologically different from non-viable cells in the same sample (**Figure 5D**). The number of injured cells was very low after drying in all the replicates/strains, possibly because the injured cells were either not able to survive desiccation, or recovered after rehydration.

Statistical evaluation of the proportion of dead cells in the samples revealed that treatments (especially nitrogen depletion) prior to desiccation (85% RH) significantly improved desiccation survival (ANOVA for repeated measures, interaction Time × Treatment, *F* = 134.61, *p* < 0.001), i.e., the number of dead cells was the lowest after the nitrogen starvation treatment, followed by the low temperature treatment. Low temperature treatment prior to desiccation (85% RH) also significantly improved survival compared to control (ANOVA for repeated measures, interaction Time × Treatment, *F* = 14.68, *p* < 0.001) when tested separately. No significant difference was found in the response of particular strains to desiccation during the whole experiment (ANOVA for repeated measures, interaction Time × Strain, *F* = 0.05, *p* = 0.95) and no significant difference between strains was found in the effect of treatment prior to desiccation on their survival (ANOVA for repeated measures, interaction Time × Strain × Treatment, *F* = 0.45, *p* = 0.78); this means that all the strains responded similarly to desiccation as well as to pre-desiccation treatments.

## Discussion

Although *Microcoleus* species inhabit water-deficient habitats (Pentecost and Whitton, 2012), no attempts have been made to determine whether they are able to survive complete desiccation. This ability gives a great advantage to organisms inhabiting arid regions and periodically dry environments. Complete desiccation is commonly defined as water loss to a content which is below 0.1 g g^-1^ dry biomass (Alpert, 2006) This important threshold corresponds to the minimum amount of water needed to form a monolayer around cell proteins and membranes (Alpert, 2006). Upon removal of this water, cells of most organisms sustain lethal damage due to irreversible changes in the native structure of dehydrated membranes and proteins as well as chemical cross-linking between proteins, sugars, and nucleic acids (Potts, 1994). The ability to survive such an extensive water loss is termed anhydrobiosis, which is a rare property among organisms (Alpert, 2006). The upper limit for complete desiccation (i.e., 0.1 g of water g^-1^ dry biomass) is roughly equivalent to air dryness at 50% RH and 20∘C (Alpert, 2005) or 30–40% RH (Sun, 2002), i.e., conditions that readily occur in terrestrial cyanobacteria habitats, especially in arid regions. Natural rates of desiccation often lead to even more extreme water loss; in hot deserts dry mass water content may drop to only 5% (ca. 0.05 g of water g^-1^ dry biomass) or less (Belnap, 2003).

Among cyanobacteria, only *Nostoc* and *Chroococcidiopsis* have been shown to withstand complete desiccation (Hawes et al., 1992; Billi and Potts, 2002). These species often co-exist with *Microcoleus* species in both hot and cold deserts (Hawes et al., 1992; Wynn-Williams, 2000; Jungblut and Hawes, 2005), e.g., as a part of soil crust communities (Belnap, 2003). However, we found that none of the *Microcoleus* strains were able to tolerate complete desiccation, even when exposing them to low temperature and nitrogen starvation prior to desiccation. Our results suggest that *Microcoleus* species lack the ability to tolerate complete desiccation to 0.03 g of water g^-1^ dry mass (as tested in this study), unlike *Nostoc*, which survives in a completely dry state for decades with only 0.02 g of water g^-1^ dry mass (Billi and Potts, 2000). There are no data available whether the amount of water below the 0.1 g of water g^-1^ dry biomass threshold affects desiccation survival of cyanobacteria. However, we assume that *Microcoleus* species, which failed to survive desiccation at 10–13% RH, would also not tolerate desiccation at higher RH values, which cause the removal of a monomolecular layer of water (i.e., up to 30–50% RH). That is because either partial or extensive removal of the monomolecular water shell around biomolecules requires cells to possess fundamentally different adaptations, e.g., replacement of water with non-reducing sugars (Crowe et al., 1990, 2002; Potts, 1994). In our experiments, a consistent drying rate of the thin biofilms was a very important condition for studying the tolerance of strains to complete desiccation. Drying of thick material would result in higher hydration of the inner layers of biomass, since the outer dry layers might provide a physical barrier against evaporation. Drying of thin biofilms insured against a false interpretation of desiccation tolerance, because even a very small increase in cell water content might be critical for cell survival.

Nevertheless, the results show that *Microcoleus* strains are able to survive extensive though incomplete dehydration to only 0.23 g of water g^-1^ dry biomass. Their resistance is not a constitutive property since cultures in their active phase of growth under optimal conditions failed to survive this drying treatment. We found that resistance to dehydration is inducible by prior exposure to suboptimal conditions, to some extent by low temperature, and to a greater extent by nitrogen starvation.

It is known that both freezing at natural rates (<10∘C min^-1^) and desiccation stresses result in loss of intracellular water, therefore protective mechanisms to these stresses frequently overlap (Mazur, 1984; Holmstrup et al., 2002; Tashyreva and Elster, 2012). Thus, this may explain why transfer of the cultures to low temperature might promote their acclimation not only to freezing, but also to drying, and act as a direct inducer of desiccation resistance. The observed patchiness of viable biomass after the drying treatment, and the absence of viable cells in some of the replicates, may be explained by partial acclimation to dehydration, which allowed for the survival of cells only in denser parts of biofilms, which presumably contained slightly more water.

It has been previously widely discussed that cultures of heterotrophic bacteria and yeasts that entered a stationary phase of growth and starved cultures display enhanced resistance to heat shock and osmotic stress, and better survive freezing and desiccation (Gilbert et al., 1990; Jenkins et al., 1990; Morgan et al., 2006; Welch et al., 2013). The stationary phase is associated with a complex of stress factors, depending on the specific conditions. However, it is commonly accepted that nutrient starvation is one of the main inducers for transition of bacterial cultures into a stationary phase of growth (Siegele and Kolter, 1992; Gefen et al., 2014). The lack of nutrients triggers not only a response directed to cope with starvation, but also a general stress response that provides cross-protection against different kinds of environmental insults (McCann et al., 1991; Siegele and Kolter, 1992). Moreover, starved cells often have a higher rate of stress tolerance than cells pre-adapted to a particular stress by exposing cells to non-lethal levels of the stress factor. For example, a short starvation episode provided *Escherichia coli* with stronger osmotic resistance than treatment with hyperosmotic solutions (Jenkins et al., 1990). Apparently, nitrogen starvation, apart from other stationary phase stresses, plays a key role in the acquisition of desiccation tolerance by cyanobacteria.

Microorganisms are able to survive desiccation conditions through avoidance of water loss (including forming spores that retain water), or true desiccation tolerance by surviving extensive water loss (Potts, 1994; Holzinger and Karsten, 2013). Desiccation tolerance is generally defined as the ability to survive severe water loss; however, there is no clear threshold between desiccation tolerant and sensitive organisms (Walters et al., 2002). Some authors suggest that desiccation tolerant organisms are able to survive dehydration below critical points of 0.25 and 0.3 g of water g^-1^ dry mass, at which point the hydration shell of molecules is gradually lost (Hoekstra et al., 2001), or loss of up to 95% of their initial water (Toldi et al., 2009). Since the studied strains failed to survive complete desiccation, it seems that *Microcoleus* species are not truly anhydrobiotic, but evidently tolerant to milder desiccation rates. Therefore, we consider that their survival strategy is attributed to tolerance of extensive dehydration, which is induced by suboptimal conditions, but avoidance of complete desiccation. *Microcoleus* avoids complete desiccation through active migration of organisms to more hydrated conditions (Garcia-Pichel and Pringault, 2001), retention of water by mucilage EPS sheaths (Chen et al., 2012) as well as by formation of thick multilayered mats that decreases the surface-to-volume ratio. In addition, such population structures generate heterogeneous conditions, under which different parts may be limited with light and nutrients. It may stimulate higher stress resistance in some of the cells. Hence, those cells might also be responsible for survival of even sudden desiccation episodes.

The three studied *Microcoleus* strains isolated from terrestrial habitats of the Arctic showed strikingly similar patterns of their response to drying. However, the results of this study do not rule out the possibility that other *Microcoleus* species/strains from extreme habitats (e.g., hot or cold deserts) or aquatic environments would respond to desiccation differently. Stronger desiccation tolerance might be induced by a stress factor other than nitrogen limitation, e.g., lack of another nutrient(s), changes in the light regime, slow dehydration, or by a combination of all the mentioned factors. Nevertheless, the results of this study provide an important background for further research on *Microcoleus* desiccation tolerance.

## Conflict of Interest Statement

The authors declare that the research was conducted in the absence of any commercial or financial relationships that could be construed as a potential conflict of interest. The Review Editor Daniela Billi declares that, despite having collaborated with author Daria Tashyreva, the review process was handled objectively and no conflict of interest exists.
