# Supplementary figures and images for: Effect of nitrogen starvation on desiccation tolerance of Arctic Microcoleus strains (cyanobacteria)
Source: Front Microbiol. 2015 Apr 8;6:278. doi: 10.3389/fmicb.2015.00278 (PMC4389727; doi:10.3389/fmicb.2015.00278)

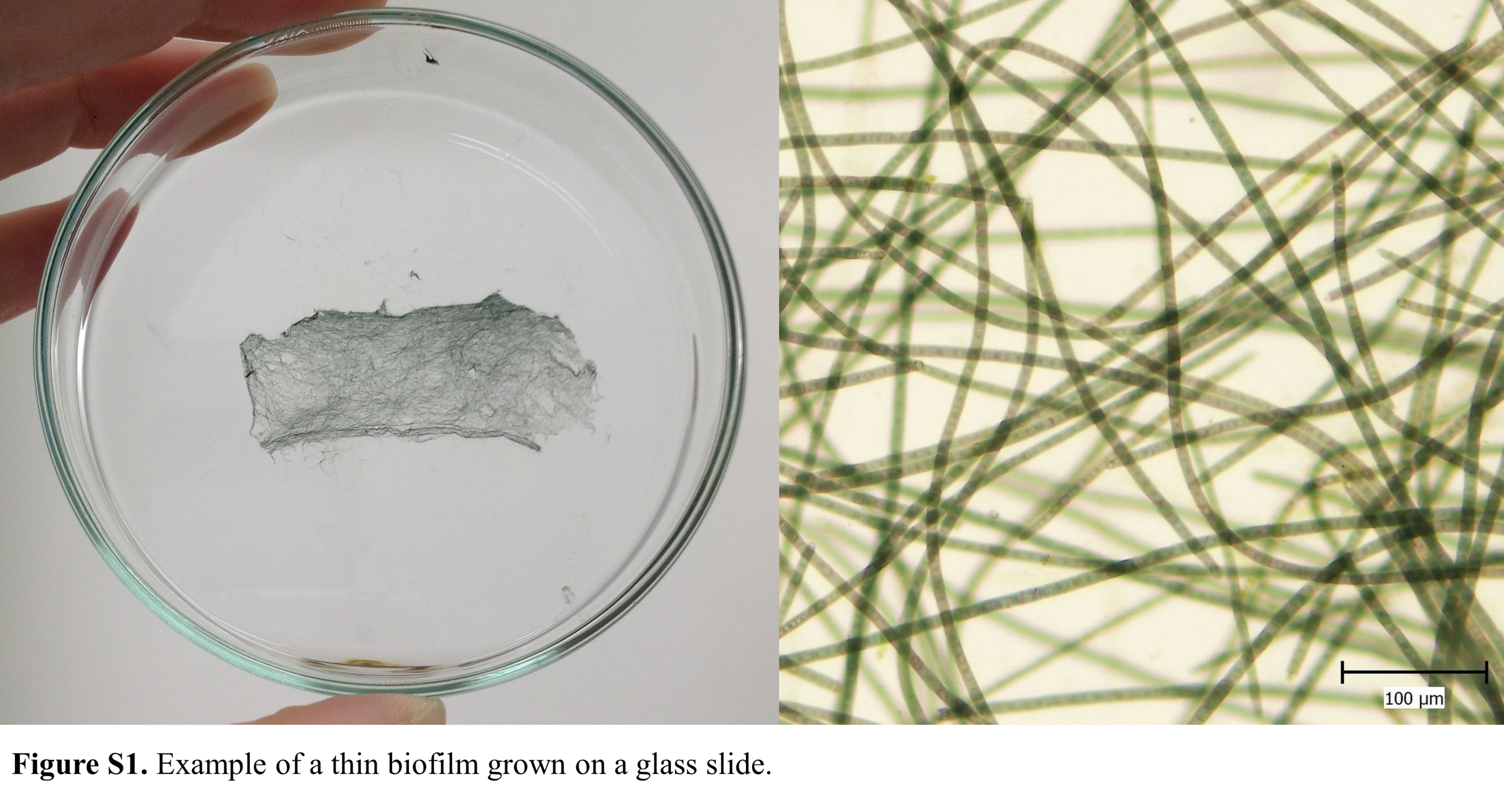

Supplement: Supplementary file 1 [file image_1.tif]
